# Integrated machine learning identifies a cellular senescence-related prognostic model to improve outcomes in uterine corpus endometrial carcinoma

**DOI:** 10.3389/fimmu.2024.1418508

**Published:** 2024-06-27

**Authors:** Changqiang Wei, Shanshan Lin, Yanrong Huang, Yiyun Wei, Jingxin Mao, Jiangtao Fan

**Affiliations:** ^1^ Department of Obstetrics and Gynecology, The First Affiliated Hospital of Guangxi Medical University, Guangxi, China; ^2^ Department of Science and Technology Industry, Chongqing Medical and Pharmaceutical College, Chongqing, China

**Keywords:** UCEC, cellular senescence, machine learning, MYBL2, CPEB1

## Abstract

**Background:**

Uterine Corpus Endometrial Carcinoma (UCEC) stands as one of the prevalent malignancies impacting women globally. Given its heterogeneous nature, personalized therapeutic approaches are increasingly significant for optimizing patient outcomes. This study investigated the prognostic potential of cellular senescence genes(CSGs) in UCEC, utilizing machine learning techniques integrated with large-scale genomic data.

**Methods:**

A comprehensive analysis was conducted using transcriptomic and clinical data from 579 endometrial cancer patients sourced from the Cancer Genome Atlas (TCGA). A subset of 503 CSGs was assessed through weighted gene co-expression network analysis (WGCNA) alongside machine learning algorithms, including Gaussian Mixture Model (GMM), support vector machine - recursive feature elimination (SVM-RFE), Random Forest, and eXtreme Gradient Boosting (XGBoost), to identify key differentially expressed cellular senescence genes. These genes underwent further analysis to construct a prognostic model.

**Results:**

Our analysis revealed two distinct molecular clusters of UCEC with significant differences in tumor microenvironment and survival outcomes. Utilizing cellular senescence genes, a prognostic model effectively stratified patients into high-risk and low-risk categories. Patients in the high-risk group exhibited compromised overall survival and presented distinct molecular and immune profiles indicative of tumor progression. Crucially, the prognostic model demonstrated robust predictive performance and underwent validation in an independent patient cohort.

**Conclusion:**

The study emphasized the significance of cellular senescence genes in UCEC progression and underscored the efficacy of machine learning in developing reliable prognostic models. Our findings suggested that targeting cellular senescence holds promise as a strategy in personalized UCEC treatment, thus warranting further clinical investigation.

## 1 Introduction

Uterine Corpus Endometrial Carcinoma (UCEC) stands as one of the most prevalent malignancies in gynecology. In China, its incidence ranks second only to cervical cancer (1). In 2023, an estimated 66,200 new cases and 13,030 deaths are projected in the United States (2).

The pathogenesis and classification of UCEC have garnered considerable attention in medical research. It is primarily categorized into two types based on biological characteristics and clinical behavior: Type I (estrogen-dependent) and Type II (non-estrogen dependent) endometrial carcinoma. Recent studies have further delineated it into four molecular subtypes: POLE ultramutated, microsatellite instability, copy-number stability, and p53 abnormal types (3). This molecular classification enriches our comprehension of UCEC heterogeneity and forms the basis for devising personalized treatment strategies (4).

Early-stage endometrial cancer commonly involves total hysterectomy and bilateral salpingo-oophorectomy (5), whereas in cases of advanced or recurrent endometrial cancer, surgery remains crucial but must be supplemented with systemic treatments such as chemotherapy, immunotherapy, targeted therapy, and endocrine therapy (6). Recent studies have concentrated on molecular markers like mutations in the PTEN, PIK3CA, ARID1A, and KRAS genes, prevalent in Type I endometrial cancers, which foster tumor growth and survival (7). Type II cancers often manifest mutations in the p53 gene and amplification of the HER2 gene (8). These findings aid in delineating distinct biological features and therapeutic targets for various tumor types.

Targeted therapies, including PI3K and mTOR inhibitors, have become essential in UCEC treatment, significantly improving outcomes for certain patients (9). For individuals exhibiting microsatellite instability or mismatch repair deficiencies, immune checkpoint inhibitors like PD-1/PD-L1 present novel therapeutic possibilities (10). The efficacy of these strategies highlights the significance of personalized medicine in UCEC treatment. However, challenges persist in precisely identifying eligible patients and devising novel medications.

Cellular senescence constitutes a multifaceted biological process involving alterations in gene expression, DNA damage accumulation, protein function loss, and cell cycle arrest (11). Serving as a critical tumor-suppressing mechanism, it inhibits cancer by constraining the proliferation of damaged or mutated cells (12). Nonetheless, the accumulation of senescent cells can foster tumor progression via the secretion of pro-inflammatory and pro-tumorigenic factors (13). Studies have demonstrated the pivotal roles of senescence-associated genes, such as p53, RB, and PTEN, in cancer development (11). Targeting SASP factors presents a novel perspective for certain cancer treatments (14, 15).

Studies utilizing public databases such as TCGA and Gene Expression Omnibus have pinpointed specific genes linked to the prognosis and treatment responses of UCEC (16–18). These genes can potentially serve as novel biomarkers for refining prognostic models. Currently, research on cellular senescence related to UCEC remains limited. Employing advanced bioinformatics to investigate the relationship between cellular senescence genes and UCEC is imperative for patient stratification and the identification of new therapeutic targets and immune treatment strategies.

## 2 Materials and methods

### 2.1 Data and patient collection

**Figure 1:**
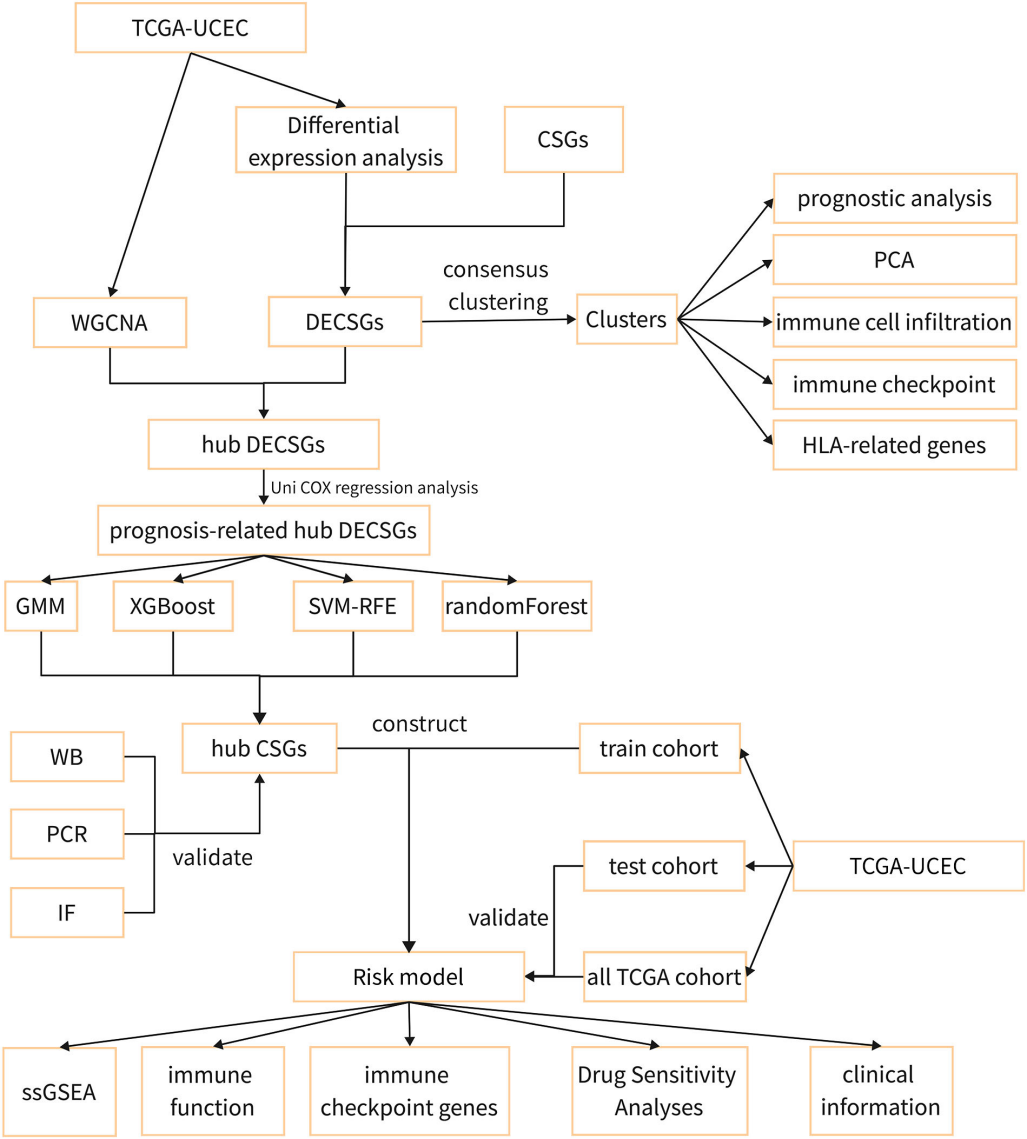
The flow diagram of the study.

**Figure 1** illustrates the methodology employed in this research. Transcriptomic and clinical data for 579 endometrial cancer patients, comprising 544 UCEC cases and 35 control subjects, were obtained from the TCGA database (https://portal.gdc.cancer.gov/). A total of 503 cellular senescence genes were sourced from the CSGene database (https://csgene.bioinfominzhao.org/index.html, **Supplementary Table 1**).

Furthermore, 20 endometrial cancer tissues and 20 non-cancerous endometrial tissues were collected from the First Affiliated Hospital of Guangxi Medical University. All UCEC diagnoses were confirmed by experienced pathologists, with pertinent clinical details provided in **Supplementary Table 2**. Following surgery, tissues were promptly transferred to a petri dish using forceps and rinsed thoroughly with physiological saline to eliminate surrounding blood clots. Subsequently, approximately 5g samples were dissected using a surgical blade for subsequent RT-qPCR and Western blot experiments. Additionally, roughly 10g of tissue was placed in 4% paraformaldehyde fixative, fixed for 24 hours, and then subjected to dehydration and paraffin embedding for sectioning. The study received ethical approval (No. 2023-S033–01), and all participants provided informed consent before undergoing surgery.

### 2.2 Differential expression analysis

In this study, we utilized the “limma” package (19) in R software to perform differential expression analysis on the UCEC dataset. The filtering criteria were set as: |log_2_FoldChange|≥1.5, and *P*<0.05. Subsequently, the differentially expressed genes and cellular senescence genes were intersected to yield a series of Differentially Expressed Cellular Senescence Genes (DECSGs).

### 2.3 Consensus clustering and subtype analysis

To identify UCEC subtypes associated with DECSGs, we utilized the “ConsensusClusterPlus” R package for consensus clustering analysis (20). This approach evaluated consistency across multiple clustering runs to determine a more stable final clustering structure, commonly employed in data analysis and bioinformatics. The clustering criteria were as follows: enhanced correlation within subtypes post-clustering, and weakened correlation between subtypes. We ensured the reliability of our results through 1,000 iterations and utilized the Probably Approximately Correct (PAC) method to determine the optimal number of clusters. Specifically, the PAC method initially generated a set of random datasets and conducted cluster analysis on these datasets to obtain a range of random cluster numbers. The PAC value quantified the dissimilarity between observed clustering results and random clustering results. A higher PAC value indicated greater dissimilarity between the observed clustering structure and random results, indicating a more robust and reliable clustering structure.

Subsequently, we employed principal component analysis (PCA) to discern variations in gene expression patterns among the clusters. Additionally, we conducted differential expression analysis across the clusters and utilized the “ClusterProfiler” (21) and “org.Hs.eg.db” packages to explore potential biological mechanisms through Gene Ontology (GO), Kyoto Encyclopedia of Genomes (KEGG), and Gene Set Enrichment Analysis (GSEA). Furthermore, the “survival” and ‘‘survminer” packages (22) were utilized to analyze the overall survival (OS) and progression-free survival (PFS) rates across the different clusters. The tumor microenvironment (TME) of endometrial cancer was assessed using the “estimate” package to understand its characteristics deeply. Based on the “CIBERSORT” package (23), we analyzed the infiltration levels of 22 immune cell types to identify differences in immune cell infiltration across clusters. Lastly, we investigated the expression differences in key immune checkpoint genes and human leukocyte antigen (HLA)-related genes between clusters. This exploration aimed to elucidate mechanisms by which tumors evade immune surveillance, providing valuable insights for the development of novel immunotherapeutic strategies.

### 2.4 Co-expression network construction

WGCNA was conducted using the “WGCNA” package (24) to construct a scale-free network associated with clinical phenotypes. The process commenced with hierarchical clustering to filter the cases, followed by the selection of an appropriate soft threshold to construct a weighted adjacency matrix. This matrix was then transformed into a topological overlap matrix (TOM), represented with colors and module eigengenes. Additionally, the Pearson correlation coefficient between the module eigengenes and clinical features was calculated to unveil potential links between gene expression patterns and clinical manifestations.

### 2.5 Cox regression analysis and machine learning algorithms

In this study, we intersected genes from key modules identified by WGCNA with DECSGs to pinpoint key DECSGs. Patients from the TCGA database with complete clinical information and survival times exceeding 30 days were selected for univariate Cox regression analysis to identify prognostically relevant DECSGs.

To accurately identify hub genes associated with UCEC, we employed four machine learning algorithms: GMM, SVM-RFE, Random Forest, and XGBoost. Firstly, GMM analysis was conducted utilizing the “SimDesign” package (25). This method examined the probability distribution of gene expression data and fit it to multiple Gaussian distributions, revealing complex underlying biological information. Subsequently, the SVM-RFE method (26) was implemented using the “e1071,” “kernlab,” and “caret” packages. This technique constructed a model based on SVM and optimized the feature set by recursively removing the least impactful features. Next, we employed the Random Forest algorithm via the “randomForest” package and the XGBoost algorithm using the “xgboost” package (27, 28). Random Forest is a robust ensemble learning algorithm that builds multiple decision trees and combines their predictions to enhance model accuracy and robustness, widely utilized in classification and regression tasks. XGBoost is an efficient ensemble learning algorithm that incrementally constructs decision trees and corrects errors to optimize model performance, identifying core features. The common genes identified by these algorithms were determined to be the core DECSGs. Finally, the relationship between these core DECSGs and the prognosis of endometrial cancer was analyzed using the external survival prognosis database Kaplan-Meier Plotter (https://kmplot.com/analysis/index.php?p=background).

### 2.6 Construction and validation of the cellular senescence-relate risk score model

UCEC samples were randomly divided into a training set and a testing set at a ratio of 7:3. Based on the expression of key DECSGs, a prognostic model was constructed within the training set using the LASSO Cox regression method.

This methodology entails an initial fitting of gene expression data and survival time via LASSO regression, followed by cross-validation utilizing the “cv.glmnet” function. Subsequently, the “coef” function is utilized to extract and compute the weights of the selected genes within the model. The model predicts patient survival prognosis through the calculation of a risk score, formulated as: Risk score = Σ (Xi*Yi), where X represents the coefficient of each gene in the model, and Y denotes the expression level of the corresponding gene. Within the training set, UCEC samples were stratified into high-risk and low-risk clusters based on the risk score. Kaplan-Meier survival analysis was employed to compare the OS between these groups, thereby validating the performance of the risk score model. ROC curve analysis, facilitated by the “timeROC” package (29), was conducted to assess the model’s accuracy in predicting patient survival rates. Finally, the model’s accuracy was further validated utilizing the independent testing set from TCGA, as well as the entire TCGA dataset.

### 2.7 Differences in immune characteristics and molecular biology between the high-risk and low-risk groups

Using the “GSEABase” and “GSVA” packages, we analyzed the infiltration fractions and immune-related functions of tumor-infiltrating immune cells in UCEC cases. Differences in immune cell infiltration between low-risk and high-risk groups were compared employing the Wilcoxon test. Moreover, the correlation between the risk score and the expression levels of immune checkpoint genes was investigated using Pearson correlation coefficients. Furthermore, comparisons of risk scores across different stages, grades, and subgroups were conducted to assess the prognostic value of the risk score.

### 2.8 Drug sensitivity analyses

To investigate the association between chemotherapeutic responsiveness and the risk score model, we employed the “oncoPredict” package (30), leveraging data from the Genomics of Drug Sensitivity in Cancer (GDSC) database (www.cancerRxgene.org). This enabled an analysis of drug sensitivity. Subsequently, we conducted comparative analyses of IC50 values across two distinct groups to assess differential therapeutic outcomes, with the aim of identifying potentially efficacious drugs for the treatment of UCEC.

### 2.9 Reverse transcription quantitative polymerase chain reaction

Total RNA was extracted using TRIzol reagent (Takara, Japan) and reverse-transcribed into cDNA. PCR was performed using the SYBR Green Master Mix kit (Qiagen, Germany), with the expression level of glyceraldehyde 3-phosphate dehydrogenase (GAPDH) serving as the internal reference. The primer sequences were provided in **Table 1**. The experiment was conducted with at least three technical replicates. We employed the 2^-ΔΔCT^ method to calculate the relative mRNA expression levels of hub genes. A CT value difference within 0.5 between replicate wells of the same sample was considered acceptable for analysis.

**Table 1:** The primers of hub DEERGs and GAPDH.

Gene name	Primer orientation	Sequences
MYBL2	Forward	CTTGAGCGAGTCCAAAGACTG
	Reverse	AGTTGGTCAGAAGACTTCCCT
CPEB1	Forward	GTCCTCCCAAAGGTAATATGCC
	Reverse	TGCAGAGCACCGACAAACA
GAPDH	Forward	CAGGAGGCATTGCTGATGAT
	Reverse	GAAGGCTGGGGCTCATTT

### 2.10 Western blotting

Cells and clinical samples were lysed with RIPA lysis buffer (Solarbio, China), and the protein concentrations were quantified with a BCA protein quantification kit (NCM Biotech, China). The protein samples were then loaded onto a 10% SDS-PAGE gel for electrophoretic separation, followed by transfer to PVDF membranes (Millipore, USA). After blocking with 5% BSA (Solarbio, China) for 1 hour, the membranes were washed three times with Tris-buffered saline containing 0.1% Tween-20 (TBST), with each wash lasting 5 minutes. Next, the PVDF membrane was incubated overnight at 4°C with specific primary antibodies (anti-β-actin, Sigma, USA, 1/10000; MYBL2, Abcam, UK,1/1000; CPEB1, abways, China, 1/1000). The following day, the membrane was incubated for 1 hour at room temperature with HRP-conjugated goat anti-rabbit IgG. Finally, the target protein band was visualized by laser scanning (Thermo Fisher, USA).

### 2.11 Immunofluorescence assay

Clinical samples were prepared into slides and deparaffinized in xylene, followed by rehydrated in 100% ethanol and sequentially dehydrated in 95%, 85%, and 75% ethanol concentrations. Antigen retrieval was carried out using sodium citrate in a microwave. To block endogenous peroxidases, the samples were treated with 3% hydrogen peroxide (H2O2), followed by incubation in a 3% Bovine Serum Albumin (BSA) solution (Solarbio, China) for blocking purposes. Subsequently, the tissues were incubated with primary antibodies (MYBL2, Abcam, UK, 1/200; CPEB1, abways, China, 1/200)) overnight at 4°C. After the primary antibody incubation, the tissues underwent incubation with secondary antibodies (Goat Anti-Rabbit IgG H&L/AF555 and Goat Anti-Mouse IgG H&L/AF488) for 1 hour at room temperature. DAPI (Solarbio, China) was added, and the samples were briefly incubated before being washed with phosphate-buffered saline. Finally, images were acquired at 400-fold magnification using a confocal microscope (Nikon AIR, Japan).

### 2.12 Statistical analysis

Data processing, analysis, and visualization were conducted using R (version 4.3.0) and GraphPad Prism (Version 9.4). Differential analysis in R was primarily conducted utilizing the “limma” package. Visualization of data was predominantly achieved through the “ggplot2”, “ggpubr”, and “enrichplot” packages. Time-dependent ROC curves were calculated and plotted using the “timeROC” package, facilitating comparisons between different models. Statistical comparisons of experimental results between different groups were executed using the Wilcoxon test, with statistical significance set as a p-value of less than 0.05.

## 3 Results

### 3.1 Identification of different expression cellular senescence genes

**Figure 2:**
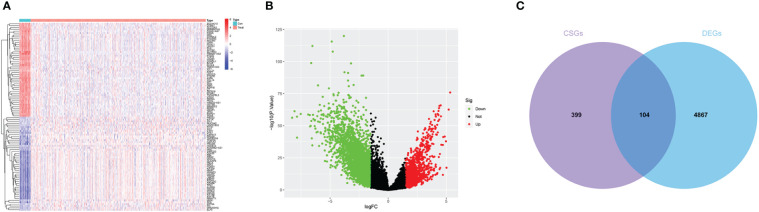
**(A, B)** The heatmap and volcano plot of differential analysis. **(C)** Intersection map of cell senescence genes and differentially expressed genes.

Differential expression analysis was performed on the TCGA-UCEC dataset. The findings revealed 1,132 upregulated genes and 3,839 downregulated genes in the endometrial carcinoma tissues compared to the control group (**Figures 2A, B**). Intersection analysis of differentially expressed genes with those associated with cellular senescence identified a total of 104 DECSGs (**Figure 2C**).

### 3.2 Construction and analysis of cellular senescence gene-related molecular clusters for UCEC

**Figure 3:**
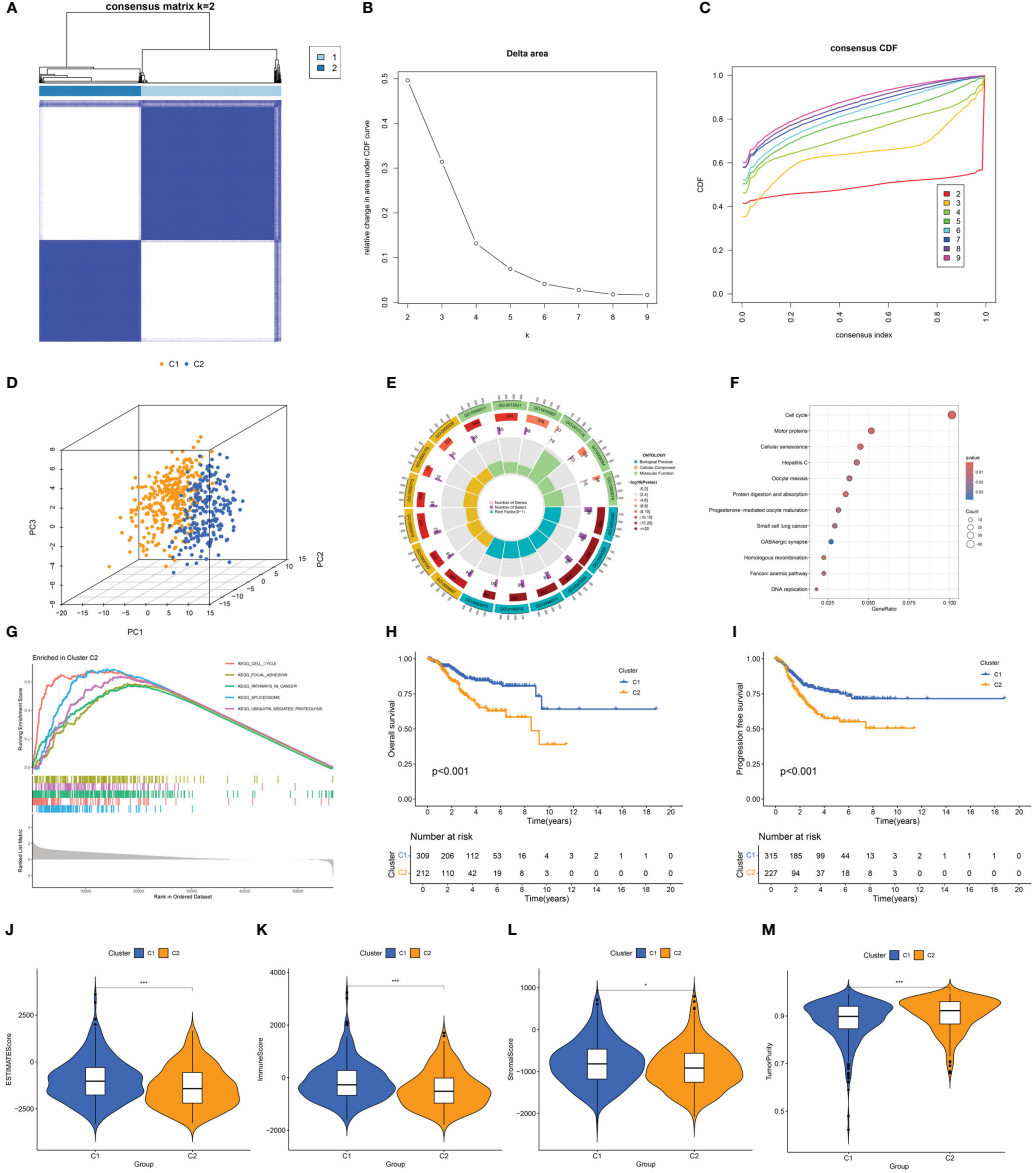
**(A)** Consensus clustering matrix when k = 2. **(B)** Relative alterations in CDF delta area curves. **(C)** Consensus CDF curves when k=2 to 9. **(D)** Three-dimensional Principal Component Analysis delineating the segregation between Cluster C1 and Cluster C2. **(E–G)** GO term enrichment, KEGG pathway analysis, and GSEA results in two clusters. **(H, I)** The difference in OS and PFS between the two clusters. **(J–M)** Differences in ESTIMATEScore, immune scores, stromal scores, and tumor purity between the two clusters (*p<0.05; ***p<0.001).

Consensus clustering was conducted based on the expression of DECSGs. As shown in the **Figures 3A–C** and **Supplementary Figure 1**, the PAC algorithm determined the optimal number of clusters to be k=2, yielding clusters denoted as C1 (n=229) and C2 (n=315). PCA affirmed the robust intergroup segregation between cluster C2 and cluster C1 (**Figure 3D**). Subsequent differential analysis of these subtypes identified 1,375 genes exhibiting differential expression. GO enrichment analysis underscored the significant involvement of these DEGs in pathways vital for nuclear division, precise chromosome segregation, and cytoskeleton functions (**Figure 3E**; **Supplementary Table 3**). Moreover, KEGG pathway analysis delineated their predominant roles in cell cycle regulation, motor proteins, cellular senescence, and protein digestion and absorption processes (**Figure 3F**). GSEA further elucidated that cluster C2 is significantly associated with pivotal biological processes encompassing the cell cycle, focal adhesion, pathways pertinent to cancer, spliceosome activity, and ubiquitin-mediated proteolysis (**Figure 3G**).

**Figure 4:**
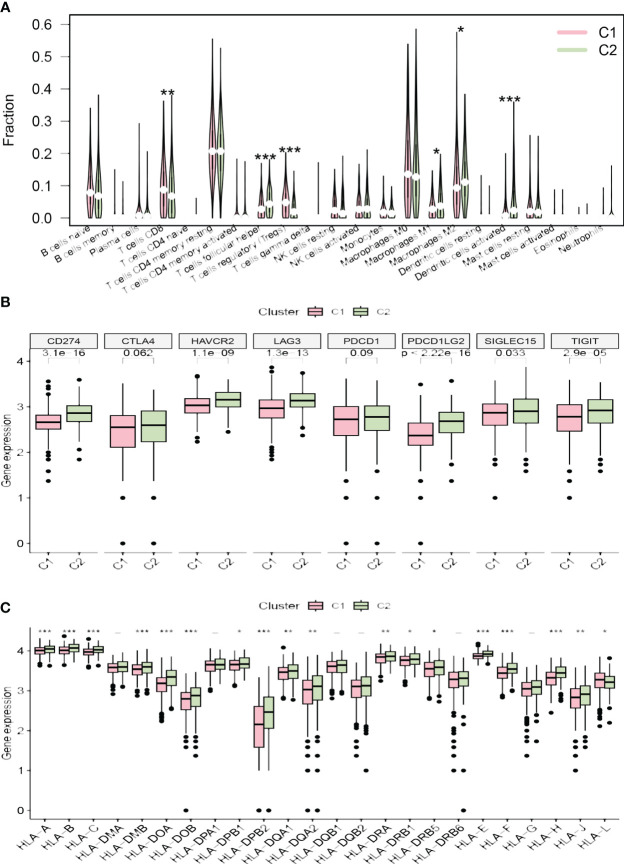
**(A)** The diagram of the difference in immune cell infiltration levels between the two clusters. **(B, C)** The different expression levels of immune checkpoint genes and HLA-related genes in two clusters, respectively (*p < 0.05; **p < 0.01; ***p < 0.001).

Survival analysis between the clusters revealed that patients in cluster C2 exhibit a shorter OS and PFS compared to those in cluster C1 (**Figures 3H, I**). Analysis of the tumor microenvironment indicated that cluster C2 demonstrates lower immune scores, stromal scores, and ESTIMATE scores, alongside higher tumor purity (**Figures 3J–M**). Further exploration of the immune landscapes among UCEC patients in the two clusters involved calculating the relative proportions of immune cells using the CIBERSORT algorithm. In comparison to cluster C1, cluster C2 exhibited significantly elevated levels of infiltration by follicular helper T cells, M1 macrophages, M2 macrophages, and activated dendritic cells, while levels of CD8 T cells and regulatory T cells (Tregs) were diminished (**Figure 4A**).

The majority of immune checkpoint genes (CD274, SIGLEC15, HAVCR2, TIGIT, LAG3, and PDCD1LG2) were highly expressed in cluster C2, while CTLA4 and PDCD1 showed no significant statistical difference between the two risk groups (**Figure 4B**). Furthermore, the expression levels of most HLA-related genes were significantly elevated in cluster C2, with the exception of HLA-L, which demonstrated decreased expression (**Figure 4C**).

### 3.3 Screening of hub prognostic DEGs

**Figure 5:**
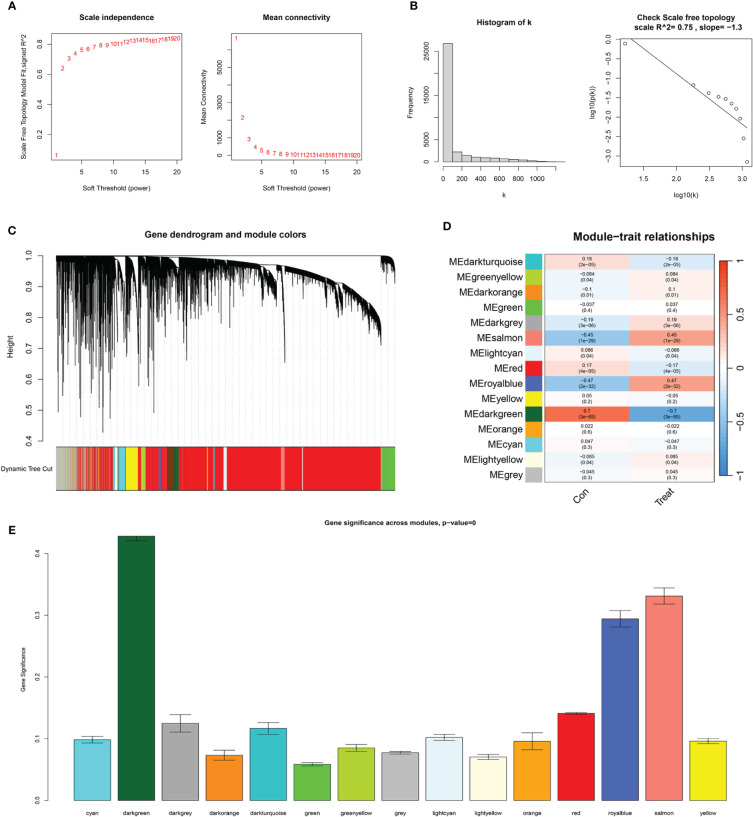
WGCNA results. **(A)** The scale-free fit index for various soft-thresholding powers (β) and the mean connectivity for various soft-thresholding powers. **(B)** Histogram of connectivity distribution and the scale-free topology when β=7. **(C)** Dendrogram of genes clustered via the dissimilarity measure. **(D)** Heatmap of the correlation between module and clinical traits. **(E)** Bar plot of gene significance across WGCNA modules.

**Figure 6:**
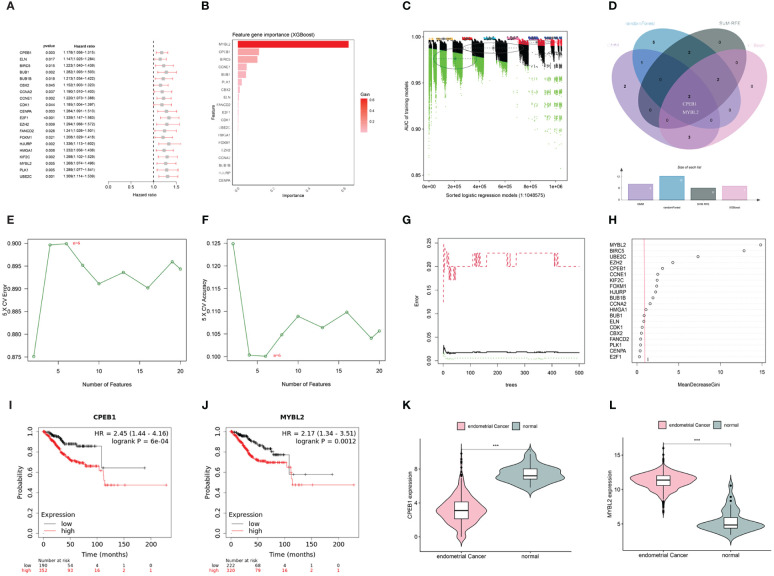
**(A)** Univariate COX analysis shows 20 genes associated with overall survival. **(B)** Screening of diagnostic biomarkers based on XGBoost algorithm (n=7). **(C)** Variable selection in GMM model (n=8); **(D)** Venn diagram of four machine learning results. **(E, F)** Through SVF- RFE algorithm selects the best biomarkers (n=6). **(G, H)** Important features selected by random forest algorithm (n=12). **(I, J)** K-M curves of CPEB1 and MYBL2 in UCEC. **(K, L)** Violin plot show the expression levels of CPEB1 and MYBL2 in TCGA-UCEC cohort(***p < 0.001).

In the WGCNA, a β value of 7 (R^2^ = 0.75) was chosen to construct a scale-free network (**Figures 5A–C**), resulting in the identification of 15 modules (**Figure 5D**). Among these, the darkgreen, royal blue, and salmon modules exhibited the highest correlation with endometrial carcinoma and were selected as hub modules (**Figure 5E**). By intersecting the WGCNA results with DECSGs, 40 critical genes were identified. Through univariate Cox regression model analysis, 20 DECSGs that displayed prognostic significance were singled out (**Figure 6A**). Further refinement was conducted using machine learning algorithms to identify hub prognostic DECSGs from these 20 genes, ensuring a more focused selection of genes with significant prognostic value. The XGBoost algorithm ultimately identified 7 central genes with a Gain > 0.01 (**Figure 6B**). In the GMM regression analysis, after 2^20^ iterations for 20 genes, the model with the highest accuracy (AUC=0.99) was determined, comprising 8 key genes (**Figure 6C**). In the SVM-RFE process, the classifier error was minimized when the number of signatures was reduced to 6; thus, these 6 genes were identified as central signatures (**Figures 6E, F**). The Random Forest algorithm, by integrating multiple decision trees, ultimately identified 12 genes with importance scores >1.0 as central features (**Figures 6G, H**). The intersection of these selected feature genes identified CPEB1 and MYBL2 as hub prognostic DECSGs (**Figure 6D**). Survival analyses from the Kaplan-Meier Plotter database revealed a significant decrease in OS of patients with endometrial carcinoma as the expression levels of CPEB1 and MYBL2 increased (**Figures 6I, J**). Compared to the control group, the expression of MYBL2 was upregulated in endometrial carcinoma, whereas CPEB1 expression was downregulated (**Figures 6K, L**). ROC curve analysis showed the areas under the curve (AUC) values for CPEB1 and MYBL2 are 0.979 and 0.974, respectively, indicating excellent diagnostic value for UCEC (**Supplementary Figure 2**).

### 3.4 Development and validation of a novel cellular senescence-related prognostic model

**Figure 7:**
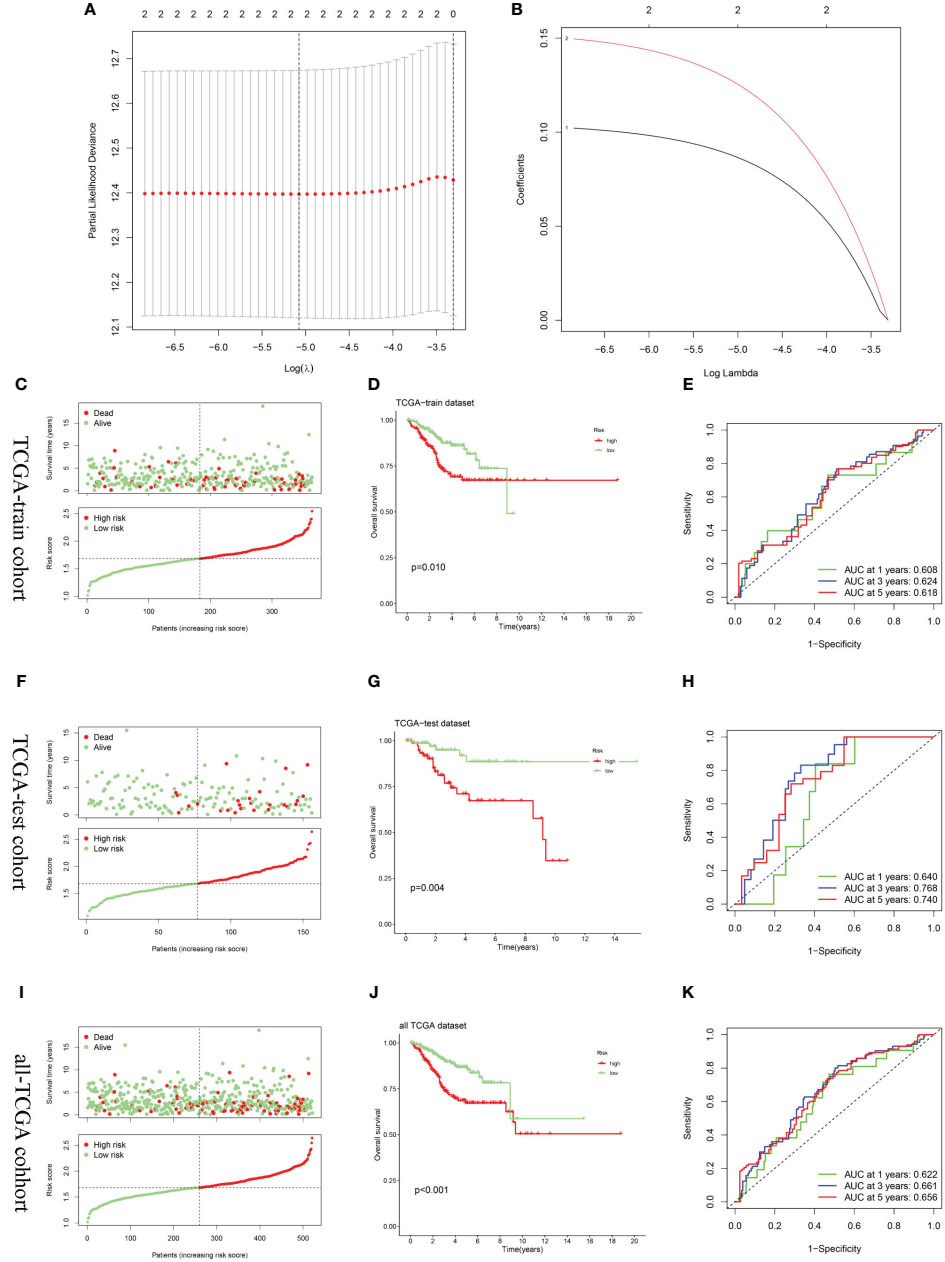
Construction and validation of the risk score model. **(A, B)** Constructed a prognostic model in the TCGA-train cohort through LASSO COX regression analysis. **(C, F, I)** Risk scores distribution and survival status of each patient in the TCGA-train cohort, TCGA-train cohort, and all-TCGA cohort, respectively. **(D, G, J)** Kaplan–Meier curves for the OS of the two subtypes in the TCGA-train cohort, TCGA-train cohort, and all-TCGA cohort, respectively. **(E, H, K)** ROC curves illustrated the predictive efficacy of the risk score for 1-, 3-, and 5-year survival in the TCGA-train cohort, TCGA-train cohort, and all-TCGA cohort, respectively.

After random division, the TCGA training set included 355 patients, while the testing set comprised 156 patients. Utilizing CPEB1 and MYBL2, a risk model incorporating two hub gene risk features was developed through LASSO Cox regression analysis in the TCGA training set (**Figures 7A, B**). The risk score was calculated as follows: Risk score = (0.1279 × expression of MYBL2) + (0.0879 × expression of CPEB1). UCEC patients were then categorized into high-risk and low-risk groups based on the median risk score. **Figures 7C, F, I** showed the distribution of risk scores and survival times across the training cohort, testing cohort, and the entire TCGA cohort. Survival analysis results demonstrated a positive correlation between higher risk scores and increased mortality in the training cohort, test cohort, and the entire TCGA cohort. According to Kaplan-Meier analysis, the overall survival of the high-risk group was significantly shorter than that of the low-risk group, indicating a worse prognosis for the high-risk group (**Figures 7D, G, J**). ROC curves demonstrated that the AUC for the 3-year time-dependent ROC for the three cohorts were 0.624, 0.768, and 0.661, respectively, indicating that the prognostic model exhibits good predictive performance (**Figures 7E, H, K**).

### 3.5 Evaluation of TME and drug sensitivity between the two risk score groups

**Figure 8:**
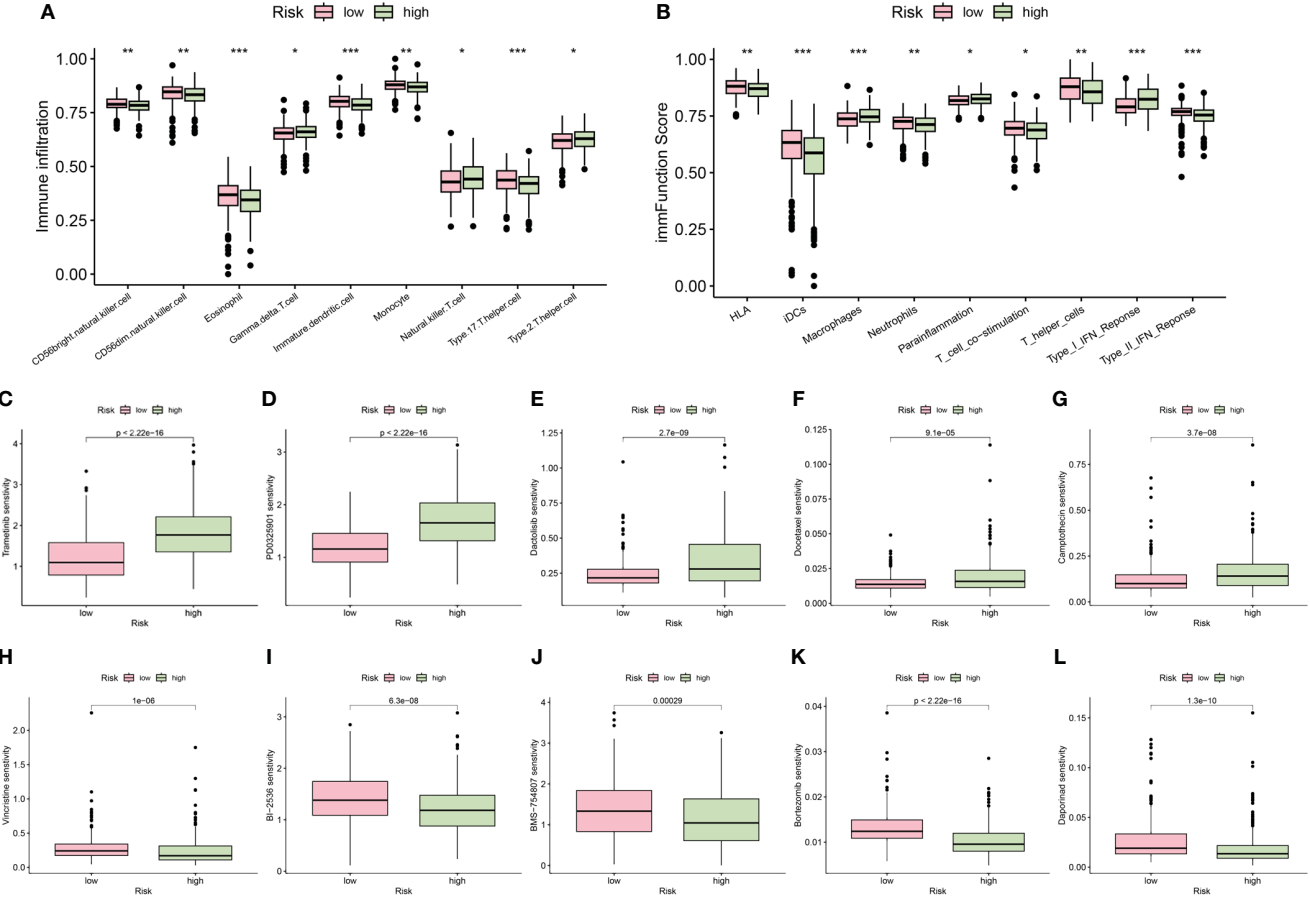
The differences of immune infiltrating cells **(A)** and immune function **(B)** between high- and low- risk groups. **(C–L)** Chemotherapy and immunotherapy sensitivity prediction between the low-risk and the high-risk groups (*p < 0.05; **p < 0.01; ***p < 0.001).

The results obtained from the ssGSEA algorithm revealed distinctive immune infiltration patterns between the high-risk and low-risk groups. Specifically, compared to the low-risk group, the high-risk group exhibited a unique immune infiltration pattern characterized by significantly lower abundance of most tumor-infiltrating immune cells, except for natural killer cells (**Figure 8A**). Regarding immune function activity, apart from macrophages and parainflammation, most immune functions were significantly higher in the low-risk group compared to the high-risk group (**Figure 8B**).

Additionally, our differential analysis of IC50 values between the groups revealed notable differences. Specifically, the IC50 values for Trametinib, PD0325901, Dactolisib, Docetaxel, and Camptothecin were substantially higher in the high-risk group compared to the low-risk group (**Figures 8C–G**). This suggests that patients with lower risk scores may derive enhanced benefits from these drugs. Conversely, IC50 values for Vincristine, BI-2536, BMS-754807, Bortezomib, and Daporinad were found to be lower in the high-risk group (**Figures 8H–L**), indicating that these drugs might be particularly effective for patients classified as high risk. These insights highlight the importance of risk stratification in tailoring chemotherapeutic strategies to individual patient profiles, potentially optimizing treatment outcomes.

### 3.6 Correlation of risk scores with clinical information, cellular senescence-related subtypes and immune checkpoints

**Figure 9:**
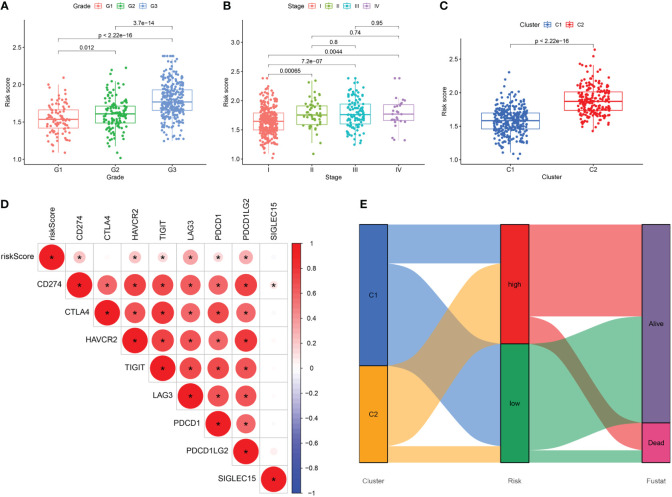
**(A–C)** The difference in risk scores between pathologic grades, clinical stages, and the two subtypes. **(D)** Correlation between the expression levels of immune checkpoint genes and risk score. **(E)** Alluvial diagram of subtype distributions and prognosis of UCEC patients. *, means p-values less than 0.05.

We conducted a comparison of risk score levels across clinical stages and grades in patients. In the TCGA-UCEC dataset, we observed that higher grades were associated with higher risk scores (**Figure 9A**). Regarding clinical stages, risk scores for patients in stages II, III, and IV were significantly higher than those in stage I. However, there were no statistical differences in risk scores between stages II, III, and IV (**Figure 9B**). Subsequently, we explored the correlation between the expression levels of immune checkpoint genes and prognostic risk scores. Notably, there was a significant difference in risk scores between the two subtypes established through cellular senescence genes (**Figure 9C**). As illustrated in **Figure 9D**, the expression of most immune checkpoint genes, except for CTLA4, was positively correlated with risk scores. An alluvial diagram illustrated the variations in cellular senescence-related clusters, risk scores, and life states (**Figure 9E**).

### 3.7 Verification of the expression of CPEB1 and MYBL2

**Figure 10:**
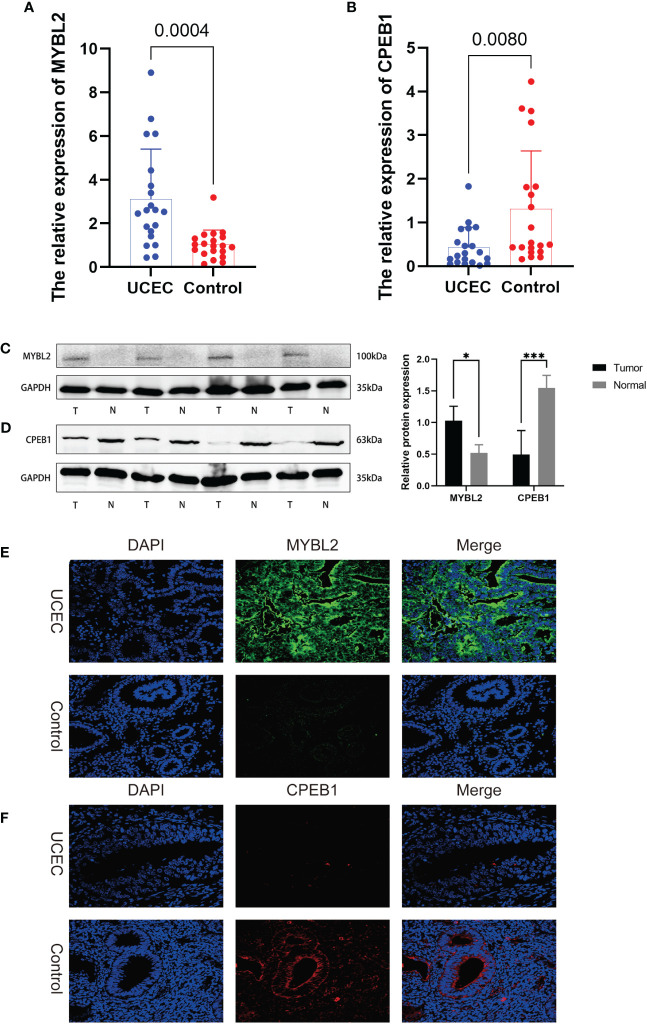
The expression levels of 2 hub genes in UCEC tissues and normal tissues were validated by RT-qPCR, WB, and immunofluorescence. **(A, B)** RT-qPCR. **(C, D)** WB assay. **(E, F)** immunofluorescence. *, and ***, means p-values less than 0.05, and 0.001, respectively.

We conducted further analysis to assess the relative mRNA and protein expression levels of the hub genes CPEB1 and MYBL2 in clinical samples. PCR results indicated that at the transcriptomic level, the relative mRNA expression of MYBL2 was significantly higher in UCEC compared to normal tissue (**Figure 10A**), while the relative expression of CPEB1 was significantly down-regulated in UCEC (**Figure 10B**). Results from WB analyses (**Figures 10C, D**) and immunofluorescence staining (**Figures 10E, F**) corroborated these findings, demonstrating that the protein expression levels of the two hub genes were consistent with the RT-qPCR results (**Figures 9A–D**).

## 4 Discussion

Uterine corpus endometrial carcinoma has been demonstrated to exhibit high levels of heterogeneity (31). The tumor microenvironment, comprising malignant, immune, endothelial, and stromal components (32), plays a pivotal role in the progression of the cancer and its sensitivity to therapeutic agents (33). The molecular attributes of endometrial cancer cells, along with the composition and dynamics of the tumor microenvironment, significantly influence these processes.

The widespread utilization of genomic sequencing has generated a plethora of biological data, offering enhanced diagnostic and prognostic capabilities across various malignancies. In recent years, researchers have developed diverse prognostic models utilizing gene expression profiles sourced from databases, employing a range of bioinformatics analysis methodologies. These models have provided valuable insights into guiding personalized treatment strategies for UCEC (34, 35).

Cellular senescence plays a crucial role in maintaining tissue stability, internal equilibrium, and serves as a natural mechanism to prevent cancer. However, under certain conditions, it can also promote tumor development (36). It has been closely associated with the onset and progression of various diseases and serves as an effective means of stratifying cancer patients (37).

Previous studies have investigated the association between cellular senescence and endometrial cancer. Gao et al. (38) conducted a bioinformatics study focusing on the role of cell senescence-related genes in UCEC and made significant progress. However, their study has certain limitations. Primarily, although they utilized various datasets from TCGA-UCEC and GEO to expand the sample size for analysis, it’s worth noting that GSE119041 dataset includes cases of undifferentiated uterine sarcoma. UCEC encompasses pure endometrioid cancer as well as carcinomas with high-risk endometrial histology, including sarcoma. Sarcomas represent uncommon subtypes with a generally poorer prognosis, and the TCGA-UCEC dataset comprises only a limited number of sarcoma cases. Incorporating data from GSE119041 into the analysis may lead to unreliable conclusions.

In our study, all samples were sourced from TCGA-UCEC, avoiding heterogeneity between diseases and samples, as well as batch effects stemming from different datasets. Unlike previous approaches that solely relied on LASSO regression to select feature genes, we employed a stepwise selection process for UCEC feature genes using methods such as WGCNA, Cox regression, and machine learning. Our findings hold promise as diagnostic and prognostic markers for UCEC. WGCNA facilitated the identification of co-expression gene modules in cancer samples, offering a refined and systematic perspective on understanding the molecular mechanisms of cancer by establishing network relationships between genes. Furthermore, the utilization of machine learning, especially in managing and analyzing large biomedical datasets, significantly enhanced the accuracy of analysis and the performance of predictive models. Leveraging these advanced algorithms allowed for the more precise identification of genes closely associated with UCEC. Lastly, we conducted multidimensional experimental validations including PCR, WB, and IF, thereby further confirming the abnormal expression of hub genes. Our study results yielded divergent findings from Gao et al., expanding the realm of research on cell senescence genes and their implications in endometrial cancer.

In this study, we conducted an in-depth exploration of the relationship between UCEC and cellular senescence genes. Utilizing 104 differentially expressed cellular senescence genes, we performed a consensus clustering analysis, ultimately categorizing UCEC into two clusters. We observed significant differences between clusters C1 and C2 in terms of biological functions, prognostic outcomes, tumor microenvironment, immune cell infiltration, immune checkpoints, and HLA gene expression. This underscores the presence of substantial tumor heterogeneity within UCEC. The KEGG results indicated that the differentially expressed genes in clusters C1 and C2 were primarily implicated in the cellular senescence pathway, highlighting the pivotal role of cellular senescence genes in UCEC. Furthermore, both KEGG and GSEA analyses indicated the activation of the cell cycle pathway.

In cluster C2, we speculated that aberrant expression of cellular senescence genes may enable damaged or potentially malignant cells to evade senescence defenses and enter a state of uncontrolled proliferation. This not only disrupted crucial cell cycle checkpoints but may also impact the expression and activity of cyclin-dependent kinases (CDKs) and cyclins, as well as their inhibitors, thereby enhancing tumor cells’ ability to override growth inhibitory signals. This propensity for unbridled proliferation facilitated the rapid expansion of cluster C2 tumor cells, exacerbating genomic instability and promoting the survival and division of DNA-damaged cells. Consequently, this promoted the malignant transformation of the C2 cluster, ultimately resulting in poor prognosis.

In the tumor microenvironment of cluster C2, we noted a higher tumor purity alongside a lower immune score. Furthermore, most of the HLA class I and class II molecules in cluster C2 were found to be upregulated. HLA class I molecules typically present endogenous antigens to CD8+ T cells, while HLA class II molecules present exogenous antigens to CD4+ T cells (39). Generally, increased expression of HLA molecules should facilitate more effective T-cell-mediated immune responses, thereby enhancing the recognition and elimination of tumor cells, ultimately improving patients’ prognosis (40). However, the results from CIBERSORT analysis revealed a decrease in the infiltration levels of CD8 T cells and regulatory T cells in cluster C2, with no significant difference observed in CD4 T cells. Conversely, the proportion of follicular helper T cells, M1 macrophages, and activated dendritic cells was found to increase.

Follicular helper T cells, primarily found in secondary lymphoid tissues, play a pivotal role in facilitating B cells interactions, thereby promoting antibody production and the formation of memory B cells (41). M1 macrophages represent an activated state of macrophages that bolster immune responses by eliminating tumor cells and pathogens (42). Activated dendritic cells capture and present antigens, thereby initiating immune responses in T cells and B cells (43). In cluster C2, combined with the upregulation of most immune checkpoint genes, these immune checkpoint molecules, typically expressed on the surface of immune cells, possessed the capacity to inhibit the activation and proliferation of T cells, fostering a tumor-promoting environment conducive to immune evasion (44). We speculated that despite adequate antigen presentation in cluster C2, the predominant influence of immune checkpoint molecules in UCEC progression renders related T cell activation ineffective. Moreover, under the influence of abnormally high expression of immune checkpoint molecules, although follicular helper T cells and M1 macrophages showed an increased proportion, their functionality may be compromised by the immunosuppressive environment, thus limiting their anti-tumor activity. Consequently, the anti-tumor immune response in cluster C2 appeared weakened, thereby facilitating tumor growth and dissemination. This underscored the potential utility of immune checkpoint inhibitors in patients within Cluster C2, as these therapeutic agents may help restore the anti-tumor immune response and impede tumor progression.

In summary, the observed upregulation of HLA genes in cluster C2, combined with the decrease in CD8+ T cells and Treg levels, alongside the heightened expression of immune checkpoint genes, revealed a complex immune regulatory network. While theoretically, this network should enhance anti-tumor immune responses, it may inadvertently lead to immune suppression due to tumor cells’ strategies for immune evasion. This phenomenon underscored the importance of emphasizing the value of immune checkpoint inhibitors in exploring immune-based therapeutic strategies for UCEC, aiming to circumvent these inhibitory mechanisms within the tumor microenvironment.

Through the application of WGCNA and Cox regression analysis, in conjunction with a series of advanced machine learning algorithms, we successfully identified CPEB1 and MYBL2 and developed a prognostic risk model. Internal validation results indicated that patients with high-risk scores exhibited significantly worse OS across the training cohort, testing cohort, and the entire TCGA cohort. Furthermore, we observed significant variations in risk scores across two clusters, clinical stages, and grades. These findings suggested that the prognostic risk model holds substantial clinical value in identifying high-risk patients.

MYBL2, a member of the MYB transcription factor family, plays a crucial role in regulating the cell cycle, particularly during DNA replication and mitosis. As a central regulator in tumorigenesis, MYBL2 is involved in the proliferation, apoptosis, and differentiation of cancer cells. Elevated expression of MYBL2 in various tumors is often associated with poor prognosis (45, 46), rendering it a potential therapeutic target in cancer treatment. As a prognostic indicator of unfavorable outcomes in osteosarcoma and a universal marker for immune infiltration across various cancers, MYBL2 exerts regulatory control over proliferation, tumor advancement, and immune cell infiltration within osteosarcoma and broader cancer contexts (47). In clear cell renal carcinoma, MYBL2 promotes malignant characteristics and impedes apoptosis through activation of the hedgehog signaling pathway (48). Within gastric cancer, MYBL2 modulates DNA damage via UBEC2 activation, thereby promoting tumor progression and resistance to cisplatin therapy (49). In ovarian cancer, the MYBL2-CCL2 axis promotes tumor progression and confers resistance to PD-1 therapy by inducing immunosuppressive macrophages (50). In colorectal cancer, MYBL2 expedites cancer progression through an interactive feed-forward activation with E2F2 (51). In our investigation, we observed upregulated expression of MYBL2 in UCEC tissues, thus suggesting its potential utility as a prognostic marker for this malignancy.

CPEB1, also known as Cytoplasmic Polyadenylation Element Binding Protein 1, exerts influence over the stability and translation of its target mRNA molecules, significantly impacting fundamental cellular processes such as growth, differentiation, and apoptosis (52). The expression and function of CPEB1 have garnered considerable attention due to its diverse expression patterns and roles across various types of cancer (53). Research into colorectal cancer metastasis has revealed a novel tumor-suppressive role for CPEB1. High methylation of the CPEB1 promoter, restricting chromatin accessibility and transcription factor binding, diminishes its expression, thereby influencing colorectal cancer progression (54). Additionally, studies have demonstrated that CPEB1 can directly target SIRT1, suppressing its translation and mediating cancer stemness *in vitro* and *in vivo*, suggesting its potential as a therapeutic target in hepatocellular carcinoma (HCC) (55). Overall, recent research has increasingly recognized the multifaceted role of CPEB1 in cellular processes and its impact on various cancers. Currently, there is a lack of research on CPEB1 in the context of endometrial cancer in the existing literature. Our analysis revealed downregulation of CPEB1 expression in endometrial cancer, a finding supported by PCR, WB, and IF assays. While we are the first to report its association with endometrial cancer, further experimental investigations are warranted to fully elucidate the underlying mechanisms.

Carboplatin, in combination with paclitaxel, has emerged as the frontline chemotherapy regimen for endometrial cancer (56). Nonetheless, substantial variability exists among patients in their responses to chemotherapy. Through drug sensitivity analysis, we have identified several drugs that hold promise for UCEC treatment. Significant differences in IC50 values of these drugs observed between distinct risk groups indicate the substantial predictive capacity of our model in predicting drug responses among patients with endometrial cancer.

Immunotherapy, particularly checkpoint inhibitors, has demonstrated high efficacy and generally favorable safety and tolerability profiles. In several clinical trials, checkpoint inhibitors have shown substantial therapeutic effects in patients with recurrent endometrial cancer, especially in those unresponsive to chemotherapy (57). Moreover, studies indicate that the use of checkpoint inhibitors can significantly enhance long-term survival rates in endometrial cancer patients characterized by specific molecular markers (58). Currently, immunotherapy drugs are increasingly being incorporated into the clinical management of endometrial cancer. PD-1 inhibitors, such as pembrolizumab and dostarlimab, have shown efficacy in treating unresectable or metastatic solid tumors with MSI-H or dMMR status. Concurrently, PD-L1 inhibitors, including atezolizumab and avelumab, are under evaluation in clinical trials for their potential in endometrial cancer therapy. Combination therapy, such as pembrolizumab combined with multikinase inhibitors like lenvatinib, is being utilized for endometrial cancer patients experiencing disease progression after prior systemic therapy. Moreover, CTLA-4 inhibitors like ipilimumab are being investigated in combination with PD-1 inhibitors to assess their efficacy in endometrial cancer treatment (59). The advent of immune checkpoint inhibitors (ICIs) has significantly transformed the therapeutic landscape for endometrial cancer, highlighting the substantial immune heterogeneity within UCEC (60). Additionally, a recent review revealed that the addition ICIs to chemotherapy can improve PFS in the overall population compared to chemotherapy alone (61). New treatment guidelines are also being formulated to explore the use of immune checkpoint inhibitors across the four molecular categories of endometrial cancer and their potential prognostic effects (62). However, not all endometrial cancer patients respond favorably to checkpoint inhibitors, particularly those with microsatellite stable (MSS) tumors or low tumor mutational burden (63). Additionally, the high costs and potential toxicities associated with these therapies limit their accessibility to all UCEC patients. Our analysis unveiled that cluster C2 exhibits elevated levels of immune checkpoint genes and a positive correlation between risk scores and immune checkpoint expression, suggesting that patients in the high-risk group may derive greater benefits from treatment with immune checkpoint inhibitors.

Our advanced bioinformatics analyses, based on a prognostic model centered on cellular senescence genes, provide novel perspectives on UCEC and present opportunities for personalized immune therapies to advance treatment strategies. Nevertheless, our study is not without limitations. Firstly, its retrospective nature and reliance on bioinformatics methodologies underscore the need for further investigations with larger patient cohorts to enhance the generalizability of the results. Additionally, while we validated the dysregulated expression of hub genes at the transcriptomic and proteomic levels, understanding their biological functions and interactions within the tumor microenvironment, particularly with regard to immune checkpoints, necessitates additional experimental exploration.

In summary, our diverse bioinformatics analyses based on senescence-associated genes have unveiled two distinct molecular subtypes of UCEC exhibiting significantly different tumor microenvironments and prognoses. Moreover, the prognostic risk model we established has demonstrated remarkable efficacy in predicting the prognosis and responsiveness to chemotherapy among UCEC patients, indicating its potential clinical applicability.

## Data availability statement

Publicly available datasets were analyzed in this study. This data can be found here: https://portal.gdc.cancer.gov/projects/TCGA-UCEC.

## Ethics statement

The studies involving humans were approved by the Medical Ethics Committee of the First Affiliated Hospital of Guangxi Medical University. The studies were conducted in accordance with the local legislation and institutional requirements. The participants provided their written informed consent to participate in this study. Written informed consent was obtained from the individual(s) for the collection of clinical specimens during the ethical approval process.

## Author contributions

CW: Conceptualization, Data curation, Methodology, Software, Visualization, Writing – original draft. SL: Validation, Conceptualization, Data curation, Formal analysis, Visualization, Writing – original draft. YH: Conceptualization, Data curation, Validation, Visualization, Writing – original draft. YW: Data curation, Validation, Writing – original draft, Investigation, Methodology, Software. JM: Software, Supervision, Visualization, Conceptualization, Writing – review & editing, Formal analysis, Methodology. JF: Funding acquisition, Supervision, Validation, Resources, Project administration, Writing – review & editing.
