# Supplementary figures and images for: Integrated machine learning identifies a cellular senescence-related prognostic model to improve outcomes in uterine corpus endometrial carcinoma
Source: Front Immunol. 2024 Jun 27;15:1418508. doi: 10.3389/fimmu.2024.1418508 (PMC11236550; doi:10.3389/fimmu.2024.1418508)

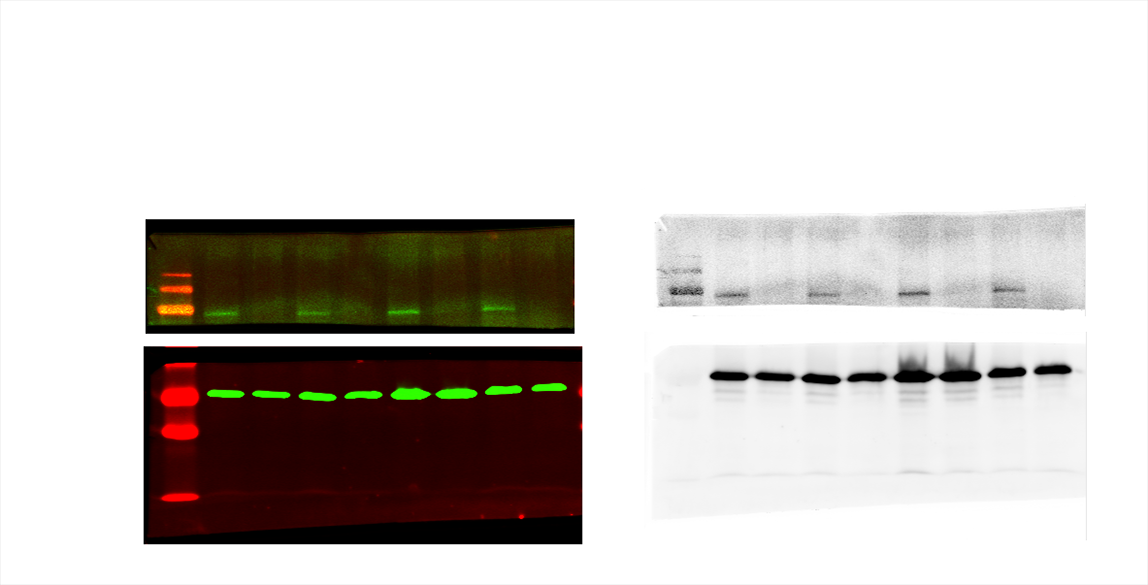

Supplement: Supplementary file 4 [file Image_1.tif]

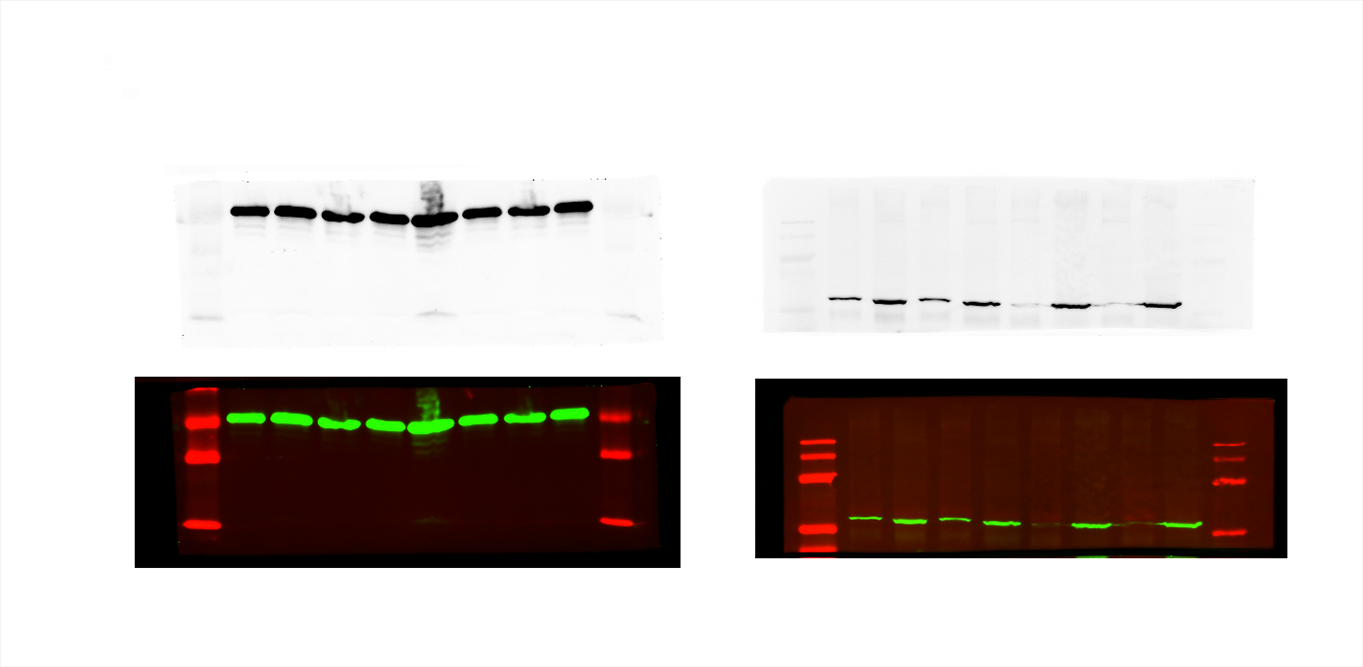

Supplement: Supplementary file 5 [file Image_2.tif]

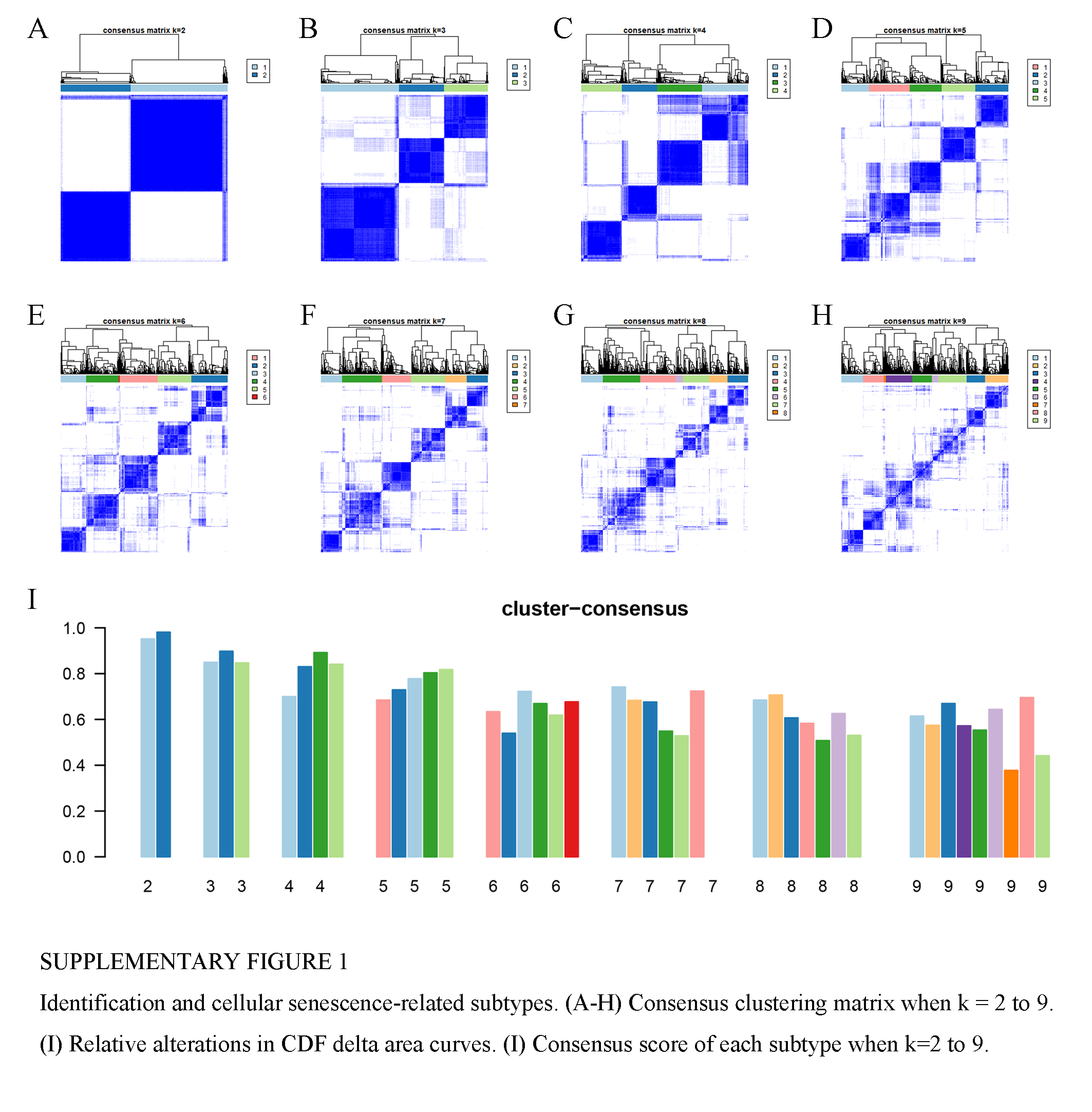

Supplement: Supplementary file 6 [file Image_3.tif]

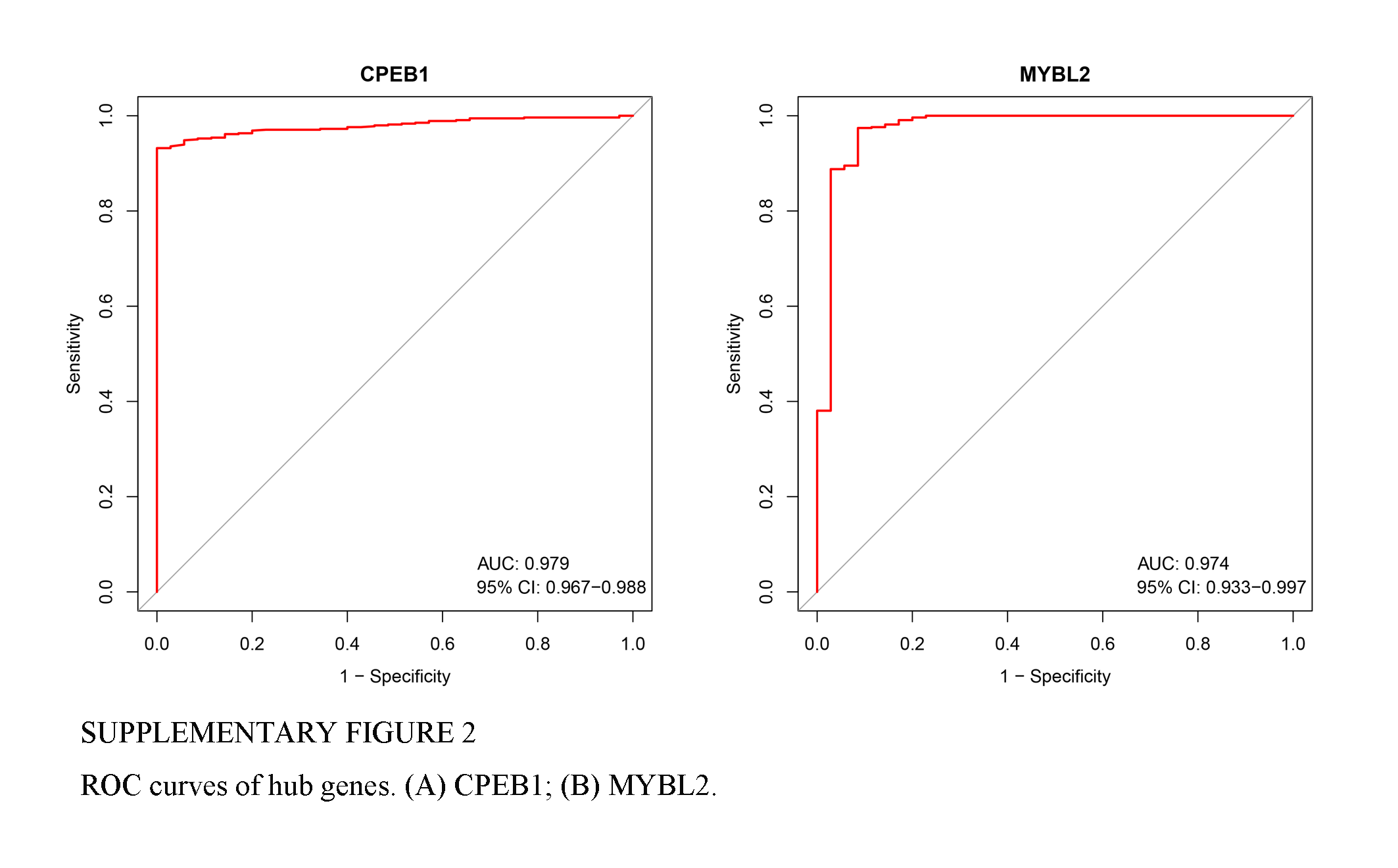

Supplement: Supplementary file 7 [file Image_4.tif]
