# Supplementary material for: Integrated machine learning identifies a cellular senescence-related prognostic model to improve outcomes in uterine corpus endometrial carcinoma
Source: Front Immunol. 2024 Jun 27;15:1418508. doi: 10.3389/fimmu.2024.1418508 (PMC11236550; doi:10.3389/fimmu.2024.1418508)
Supplement: Supplementary file 8 [file Table_1.docx]

Supplementary Table 2 The clinical data of UCEC patients.

| Clinical parameters | | N |
| --- | --- | --- |
| Age | ＜60 | 13 |
|  | ≥60 | 7 |
| Grade | G1-G2 | 14 |
|  | G3-G4 | 6 |
| Differentiation | Low and Middle | 12 |
|  | High | 8 |
| Invasion depth | Superficial | 6 |
|  | Deep | 14 |
| Lymphatic metastasis | No | 15 |
|  | Yes | 5 |
| Vascular invasion | No | 19 |
|  | Yes | 1 |
| Distal metastasis | No | 20 |
|  | Yes | 0 |
